# Electric oscillation and coupling of chromatin regulate chromosome packaging and transcription in eukaryotic cells

**DOI:** 10.1186/1742-4682-9-27

**Published:** 2012-07-03

**Authors:** Yue Zhao, Qimin Zhan

**Affiliations:** 1 State key laboratory of molecular oncology, Cancer Institute & Hospital of Chinese Academy of Medical Sciences, Peking Union Medical College, Beijing, 100021, China

**Keywords:** Oscillation cluster, Long non-coding RNA, Electromagnetic field, CTCF, HOX, Chromosome packaging, Intron

## Abstract

Transcription in eukaryotic cells is efficiently spatially and temporally regulated, but how this genome-wide regulation is achieved at the physical level remains unclear, given the limited transcriptional resources within the nucleus and the sporadic linear arrangements of genes within chromosomes. In this article, we provide a physical model for chromatin cluster formation, based on oscillation synchronization and clustering of different chromatin regions, enabling efficient systemic genome-wide regulation of transcription. We also propose that the electromagnetic field generated by oscillation of chromatin is the driving force for chromosome packing during M phase. We further explore the physical mechanisms for chromatin oscillation cluster (COC) formation, and long-distance chromatin kissing. The COC model, which connects the dots between chromatin epigenetic modification and higher-order nuclear organization, answers many important questions, such as how the CCCTC-binding factor CTCF contributes to higher-order chromatin organization, and the mechanism of sequential transcriptional activation of HOX clusters. In the COC model, long non-coding RNAs function as oscillation clustering adaptors to recruit chromatin modification factors to specific sub-nuclear regions, fine-tuning transcriptional events in the chromatin oscillation clusters. Introns of eukaryotic genes have evolved to promote the clustering of transcriptionally co-regulated genes in these sub-nuclear regions.

## Background

The regulation of eukaryotic gene transcription can be viewed as forming order out of chaos. To understand it, it is necessary to explore the physical nature of oscillating heterogeneous chromatin regions with nonlinear contacts and interactions. Recent advances in the fields of applied mathematics and physics together with technologies evolved from the application of high-throughput DNA sequencing allow us to envision nuclear chromatin organization as clustered. This knowledge provides us with new insights into the regulation of gene transcription, the functions of long non-coding RNAs and the evolution of introns in eukaryotic cells.

Lieberman-Aiden *et al*. reported that genome-wide Hi-C maps indicate a fractal globule model for chromatin conformation that enables maximally dense packing while preserving the ability to fold and unfold any genomic locus easily. They suggested spatial segregation of open and closed chromatin to form two genome-wide compartments and the presence of chromosome territories [1]. However, the physical basis for such a chromatin conformation and its functional relevance to the regulation of gene transcription were not explored in detail. We suggest that a physical model of synchronization and clustering of pulse-coupled oscillators is applicable to chromatin organization.

## Pulse-coupled chromatin oscillation clustering (COC) model

Synchronization and clustering of pulse-coupled oscillators are determined by two major factors: the natural frequencies of the oscillators and the coupling strength within a clustering compartment. At a given coupling strength, unsynchronized oscillators form multiple clusters, each of which has a synchronized average frequency. These clusters are preferentially formed among oscillators with similar natural frequencies at a given coupling strength among them. As the coupling strength increases the oscillation clusters merge with each other forming fewer and bigger clusters. Each of these has an average frequency that is shared among the synchronized oscillators within the cluster. At certain threshold coupling strength, all oscillation clusters merge into one synchronized oscillation system [2-5].

Using the pulse-coupled oscillation clustering model, eukaryotic nuclear chromatin organization can be viewed as the result of the differential clustering of oscillating chromatin regions. The coupling strengths of chromatin regions are determined by physical interactions among chromatin-associated proteins, the electromagnetic fields around the oscillating chromosomal regions, and the hydrogen and other bonds linking different chromatin regions within the same chromosome. The natural frequency of an oscillating chromatin region is determined by the physical properties of DNA-protein complexes in that region, which can be changed by its epigenetic state and the protein factors associated with it. The pulse-coupled oscillation clustering model explains the proximal enrichment effect of intra- and inter-chromatin contacts observed by Lieberman-Aiden et al. [1], as the coupling strength increases when two chromatin regions are in close proximity. The dynamic regulation of chromosome compaction can be viewed in terms of multiple orders of partial entrainment and clustering events of the oscillating chromatin regions. Primary clustering of chromosomal regions forms oscillating clusters with average frequencies that become oscillators for a higher order of synchronization and clustering [6]. During M phase, when chromatin regions are compacted into chromosomes, each chromosome can be viewed as a synchronized oscillating cluster with one average frequency, which is the physical basis of the electromagnetic field around that chromosome. During compaction, the coupling strength within the chromosome increases as a result of epigenetic modifications and the binding of chromosome compaction proteins such as condensin.

## Electromagnetic properties of duplicated M phase chromosomes

Andrews et al. have studied the effects of high frequency electric fields on mammalian chromosomes in vitro. This research confirmed that high frequency electric fields (range 2 to 50 MHz) on human and Chinese hamster chromosomes can be oriented, aligned and translated by an oscillating electrical force. They also observed that above certain threshold field strengths, the chromosomes orient themselves with their long axes along the field direction. The dependence of this threshold on frequency was measured and was found to be much greater at low than at high frequencies [7]. Using electric dichroism experiments, Crothers reported a permanent dipole moment in dinucleosomes linked with 140 and 175 base pairs of DNA [8]. Sun et al. suggested an electrostatic mechanism of nucleosomal array folding, revealed by computer simulation, that explains the salt-dependent chromatin fiber conformations [9]. Schalch et al. reported that the X-ray structure of an oligonucleosome revealed linker DNA making zigzags back and forth between two stacks of nucleosome cores, which form a truncated two-start helix and do not follow a path compatible with a one-start solenoidal helix [10]. Grigoryev et al. reported evidence for heteromorphic chromatin fibers, showing that the two-start zigzag topology and the type of linker DNA bending that defines solenoid models may be simultaneously present in a structurally heteromorphic chromatin fiber with uniform 30 nm diameter. Their data also suggest that dynamic linker DNA bending by linker histones and divalent cations in vivo could mediate the transition between tight nucleosome packing within discrete 30-nm fibers and self-associated higher-order chromosomal forms [11].

**Figure 1:**
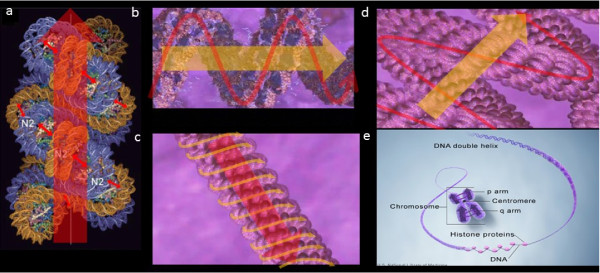
** (a) The small red arrows indicate the electric oscillations generated between the neighboring histone octamers by excitation of entropic energy within the cell nucleus.** The big red arrow represents the electric field generated by the electric oscillation along the 30 nm chromatin fiber. **(b-d)** Schematic illustration of several orders of oscillation coupling and clustering of EMFs in chromatin fibers, which facilitate the multi-step event of M phase chromosome packaging. The red and orange arrows indicate the multiple orders of EMFs generated during chromosome packaging. **(e)** The purple arrows indicate the EMFs of compacted M phase chromosome arms; the purple cycles indicate coupling of EMFs. The duplicated chromosome arms hold a juxtaposed position.

However, the physical mechanisms that regulate the higher-order chromosome packing of metaphase chromosomes have not been fully characterized. Here we present a hypothetical mechanism of chromosome compaction. The 30 nm chromatin fiber is initially formed through electrostatic forces between neighboring nucleosomes. Under intracellular stochastic energy excitation, electric dipolar oscillation would be generated between such nucleosomes. After synchronization and coupling of the oscillations, regulated oscillations are generated along the 30 nm chromatin fiber, and the oscillation coupling process further compacts the 30 nm fiber [12-14]. The compaction facilitates further packing of this fiber into the 300 nm chromatin fiber; the electric field bends according to the physical curvature of the compacting 30 nm fiber, generating an oscillating electromagnetic field that goes through the 300 nm fiber. The second round of oscillation synchronization and coupling results in the formation of the 250 nm chromosome fiber and facilitates its packing into the 700 nm chromosome fiber. The bending of the electromagnetic field of the 250 nm fiber around the curvature of the 700 nm fiber generates a higher-order electric field that goes through 700 nm fiber; thereafter, oscillation coupling further compacts the 700 nm fiber into chromosome arms [15,16]. We speculate that the source of the energy for the electric dipolar oscillation between neighboring nucleosomes in a chromatin fiber is the variety of intracellular entropic forces, and the direction of the electric oscillation primarily depends on the zigzag arrangement of neighboring nucleosomes along the 30 nm fiber (Figure 1).

Theoretically, the oscillation clustering model explains the closely-juxtaposed configuration of duplicated chromosomes during M phase, which is counter-intuitive from the perspective of electrostatic repulsions between the duplicated chromosome arms. As the homologous chromosomal regions comprise synchronized chromosomal oscillation clusters with identical natural frequencies, they tend to cluster together. The same scenario can apply to synapsis during meiosis, as the oscillations of the electric fields of homologous chromosomes match each other, preventing synapsis between non-homologous chromosomes.

## Mechanism of chromosome kissing by oscillation

Non-M phase chromatin organization is of great research interest, and attempts have been made to capture 3D chromatin organization at high resolution [17-19]. These studies have indicated that specific chromatin regions are likely to be juxtaposed in close proximity within the nucleus; the phenomenon is also known as ‘chromosome kissing’. As described above, the synchronized oscillation of oligonucleosomes along the 30 nm chromatin fiber of a particular chromosome region generates a longitudinally oscillating electric field. By binding to certain protein complexes via the consensus DNA elements, some chromatin regions are similarly epigenetically modified by various protein factors. Added to the homologies between DNA sequences, these chromatin regions therefore share similar electromagnetic frequencies and attract each other. Chromatin regions containing certain consensus DNA elements, such as the binding site CBS for the CCCTC-binding factor (CTCF) and the polycomb response element (PRE), bind to specific protein complexes to form a closely juxtaposed conformation that becomes the anchoring point for transcriptional loops. The protein factors binding to these elements are known as chromosome organizers, which organize higher-order nuclear chromatin structures. The physical scenario we have proposed here does not exclude the possibility of chromosome looping through physical interactions of protein complexes that bind to juxtaposed chromatin regions. However, the likelihood that such a mechanism regulates clustering events between distant chromatin regions is low; it is more likely to be involved in short distance chromatin looping, such as these chromosome loops observed between the promoter regions and the upstream regulation elements.[20-22] Alternatively, it is possible that the oscillation clustering of chromatin regions by EMF brings distant chromatin regions into closely juxtaposed positions, and the oscillation cluster would be further stabilized by protein complexes.

**Figure 2:**
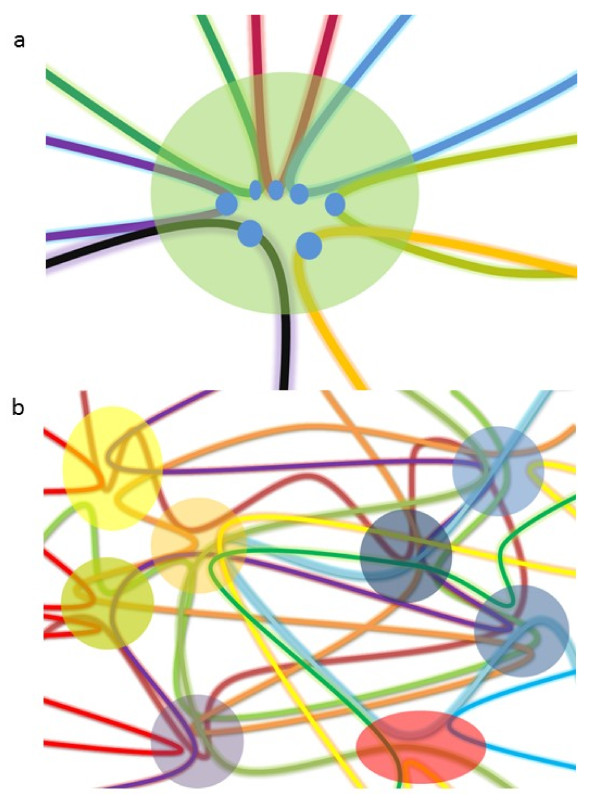
** (a) Chromatin regions that share similar natural frequencies are clustered into a proximal region within the cell nucleus.** The different colored loops represent different chromatin loops and the blue dots represent chromatin regions functioning as oscillators. These regions may share similar genomic and epigenetic features or bind to certain chromatin-associated protein complexes. They function as chromatin organizers in the nucleus. The green circular region, which contains many of the juxtaposed chromatin oscillators, represents a chromatin oscillation cluster. **(b)** Schematic illustration of how different chromatin loops are wired through different chromatin oscillation clusters. These COCs further cluster with each other on the basis of average frequencies, forming higher-order chromatin clusters.

Certain chromatin regions function as synchronized oscillators, either by coupling of the electromagnetic field generated by longitudinal oscillation of nucleosomes, or by the physical interactions of protein-DNA complexes. The density and positioning of nucleosomes in a chromosome region, the physical properties of histone octamers, as well as the protein complexes binding to the chromatin region, together determine the coupling strength of that region. Through the mechanism described above, these chromatin regions will cluster with each other if they share similar oscillation frequencies, and function as chromatin organizers to shape the higher-order chromatin structures. (Figure 2) from this point of view, we could dissect a segment of chromatin into a number of partially entrained oscillators separated by loosely-coupled chromatin regions in which the structures are less compacted. In addition, it is noteworthy that oscillation clustering would facilitate chromosome rearrangement under physiological and pathological conditions. Thus, specific epigenetic modifications of these chromatin regions would relay specific chromosome rearrangements to upstream signals, resulting in alterations of both sub-nuclear chromatin structures and chromosome structures.

## Chromatin oscillation clusters in transcriptional regulation

The chromatin oscillation cluster (COC) has a common role in transcriptional regulation of eukaryotic genes. For example, sequential transcriptional activation of the HOX gene clusters can be viewed as a sequential uncoupling event of different HOX transcriptional loops within COCs [23-27]. The uncoupling process is regulated by epigenetic modifications and chromosome remodeling of chromatin regions that are critical for the transcriptional loop formation in the HOX gene cluster, such as the PcG body binding sites. Epigenetic modifications of DNA and histones, and alterations of the chromatin-associated proteins of COC regions, change the natural frequencies as well as the coupling strengths of individual transcriptional loops within the COC, resulting in the uncoupling of a specific transcriptional loop from the COC.

Multiple lines of evidence indicate that COC formation increases the epigenetic stability of the clustered chromatin regions. CTCF binds to CBSs, which function as a chromatin barrier, creating chromosome boundaries that segregate transcriptionally active from heterochromatin regions. Through shaping higher-order chromatin structures, CTCF organizes chromosomal loop formation across the vertebrate genome. Yoon Jung Kim et al. reported that OCT4 antagonizes heterochromatin loop formation by preventing cohesin loading at CBSs, and knocking-down CTCF or over-expression of OCT4 abolishes HOXA cluster formation and up-regulates several HOXA genes [26]. Handoko et al., using chromatin interaction analysis by paired end tag (ChIA-PET) sequencing, further characterized five models of CTCF-regulated chromatin clustering in correlation with genome-wide transcriptional regulation [28]. Within the COC model, CTCF may shape the chromatin boundaries of individual oscillators in different chromatin states, and physical properties consequently contribute to COC formation.

Components of the nuclear envelop(NE) such at the nuclear pore complex(NPC) and nuclear lamina also play a role in the establishment of COC. These components specifically target certain genes to cluster at sub-nuclear territories that function as the transcription factories.[29,30] Brickner et al. reported that genes with identical zip codes cluster together in yeast. And targeting to the nuclear periphery and interaction with the nuclear pore is a prerequisite for zip codes mediated gene clustering, but clustering can be maintained in the nucleoplasm. They found insertion of two different zip codes (GRS I or GRS III) at an ectopic site induced clustering with endogenous genes that have that zip code. And transcription factor Put3 recognizes the GRS I zip code to mediate both targeting to the NPC and interchromosomal clustering.[31] Similarly, using genomic repositioning assays Zullo et al. reported lamina associated chromatin domains (LADs) spanning the developmentally regulated IgH and Cyp3a loci contain discrete DNA regions that associate chromatin with the nuclear lamina and repress transcription in fibroblasts. Fine-scale mapping of LADs reveals numerous lamina-associating sequences (LASs) enriched with a GAGA motif. This repeated motif directs lamina association and interacts with the transcriptional repressor cKrox, in a complex with HDAC3 and Lap2b. And knockdown of cKrox or HDAC3 results in dissociation of LASs/ LADs from the nuclear lamina.[32] These results reveal targeted chromatin oscillation clustering mediated by components of NE couple spatial compartmentalization of the nucleus with functional compartmentalization of the genome.

Long non-coding RNAs are known to function as scaffolds that recruit certain chromosome remodeling complexes to the promoter regions of specific genes to regulate their transcription [33]. Cavalli et al. reported that RNAi components such as AGO1, PIWI, and Dcr-2 are required for the maintenance of PcG2-mediated chromatin clusters that contribute to the co-suppression of genes within the HOX cluster [34]. RNAi components possibly maintain COC stability by recruiting heterochromatin modification complexes to maintain the epigenetic state of PCG2-binding chromatin regions, and by degrading the long non-coding RNAs in the COC, which would change the epigenetic state of the chromatin regions. Bantignies et al. reported that genomic regions of the two distant Hox complexes are clustered within nuclear PcG bodies in *Drosophila* tissues where they are co-repressed, and mutations in any of the clustered chromatin regions weaken the silencing of genes in the others. This finding indicates that HOX gene clustering and co-regulation are not restricted to the same HOX gene cluster but can span across chromatin territories more than 10 Mbp in extent [35].

## Long non-coding RNAs in the COC model

Chambeyron et al. suggest that higher-order chromatin structures regulate the expression of the HOX cluster at several levels, including locus-wide changes in chromatin structure and the temporal programming of expression of different genes [23]. Many large intergenic RNAs (Linc RNAs) genes are located within HOX gene clusters, some of which have already been well studied [36,37]. These Linc RNAs are transcribed following the initial transcription onset signal, which is also spatially and temporally regulated. After transcription, these Linc RNAs are clustered autonomously in a COC on the basis of their natural frequencies and the local coupling strengths, which are determined by specific chromatin-associated proteins and the spatial relationship between the COC and the Linc RNAs. Long non-coding RNAs functioning as oscillation adaptors for transcription factors have two advantages. First, they function as scaffolds that can recruit multiple transcription factors and chromosome modifiers to specific genome loci to allow specific epigenetic modification. Secondly, the physical properties of RNA-protein complexes are more likely to have similar natural frequencies with COC than individual proteins, which is critical for transcription factor trafficking in the nucleus.

Certain long non-coding RNAs mediate the sequential transcriptional activations of the HOX gene clusters through epigenetic modifications of their proximal chromatin domains within the COC, uncoupling the transcriptional loop from the repressive COC and thus activating transcription within the loop. After a period of transcription, the epigenetic state of the transcriptional loop is reverse-modified to a silent state because of inability to cluster in close proximity to the long non-coding RNAs recruited with transcription activators. It is possible that the uncoupling of the first transcriptional loop from the COC results in the exposure of the second transcriptional loop to the long non-coding RNAs within the region. Alternatively, long non-coding RNAs transcribed from the first transcriptional loop could cluster in close proximity to the second transcriptional loop of the COC.

So the oscillation clustering events in chromatin permit exact spatial and temporal regulation of transcriptional activators and repressors in specific sub-nuclear loci, contributing to efficient and precise sub-nuclear level transcriptional regulation. Huarte et al. reported that a P53-regulated long non-coding RNA, lincRNA-p21, is able to repress a number of pro-survival genes to mediate P53 apoptosis programs, partly through binding to the hnRNPK protein and recruiting this chromatin modifier to the promoter regions of those genes [38]. With the COC model, we can explain the phenomenon as an example of synchronized gene transcriptional regulation involving genes posited at different linear genomic locations but co-regulated through chromatin oscillation clustering.

## Evolution of introns and COC

One of the characteristics of eukaryotic genomes is the evolution of a large number of introns, which are usually much longer than exons. If introns exist only for the purpose of mRNA transcription and splicing, they are not economical for the cell. However, from the oscillation clustering point of view, introns evolved to establish specific clustering events in chromatin regions. The insertion of introns into a gene would adjust the average frequency and coupling strength of the relevant chromatin region to promote formation of a COC and allow the transcription of eukaryotic genes to be systematically regulated. Moreover, introns and intergenic sequences would facilitate the transcription of genes at the uncoupled transcriptional loops to extend out of the repressive cluster regions. Thus, transcription can take place in the sub-nuclear regions where transcriptional resources are most available, and transcriptional effects on the rest of the genes in the COC, where a repressive state is maintained, are minimized.

## Experimental verification of the hypothesis

Interesting questions arise as how to verify the COC hypothesis experimentally. From the reductionist point of view, verification could be achieved by transfection of artificial mini-chromosomes into live cell nuclei. Artificial mini-chromosomes can be specifically designed to contain key chromosome organizer motifs such as the CTCF binding site, and artificially designed introns and exons. Fragments of these mini-chromosomes can be labeled with different fluorescent labels, so their locational changes within the living cell nucleus can be observed by microscopy. In combination with chromosome conformation capture (3 C) and Hi-C techniques, we can study the genomic landscape of chromosomal regions that cluster with the artificial mini-chromosomes. Such technologies have already been developed. For instance, using the 3D two-color FISH and immunostaining technique (FISH-I), Grimaud et al. reported that an artificial mini-chromosome containing Fab-7, a well-characterized PRE-containing element, recurrently clusters with PcG bodies, which are endogenous chromosomal regions containing homologous Fab-7 copies [35]. Wenqin Wang et al. used super-resolution fluorescence microscopy in combination with a chromosome-conformation capture assay to study the distribution of major nucleoid-associated proteins in live *Escherichia coli* cells. They found that H-NS, a global transcriptional silencer, formed two compact clusters per chromosome, driven by oligomerization of DNA-bound H-NS through interactions mediated by the amino-terminal domain of the protein. H-NS sequestered the regulated operons into these clusters and juxtaposed numerous DNA segments broadly distributed throughout the chromosome. Deleting H-NS led to substantial chromosome reorganization [39]. With some modifications and the refinement of such technologies, we could systemically study the genomic features of chromosomal clustering events. However, a substantial number of experiments would be required for such work.

On the other hand, we can utilize the currently popular Hi-C techniques and bioinformatic approaches to study chromosome clusters. With such technologies, Changying Guo et al. reported two types of chromatin loops in the immunoglobulin heavy-chain gene locus; one type links chromatin regions forming the first order chromatin cluster, while the other links different chromatin clusters into a juxtaposed position forming a higher-order chromatin cluster [6]. Deep mining of Hi-C chromosomal interaction data in combination with intron-extron genomic localization, as well as the transcriptional state of the chromatin regions, is likely to yield insights that could support the COC model. It would be particularly interesting to develop physical equations that address the detailed characteristics of EMFs in chromosomal regions, and their natural frequencies. By establishing such physical equations we can link the Hi-C chromosomal interaction data with the genomic features of chromatin regions. This would provide us with the rationale as to why certain introns and inter-genetic sequences are positioned at particular locations in the chromosome. In addition, epigenetic modification of histones such as phosphorylation and methylation, which change the surface electric charge and molecular weight of those proteins, may significantly change the natural frequency of a chromatin region. So it is also important to explore the epigenetic features of chromosome oscillation clusters. Technologies such as genome-wide histone profiling and Hi-C chromosome interaction analysis could be used in combination to address these issues [40].

## Conclusion

Intracellular electric fields remain an obscure subject to the majority of biologists, as there are still many technological barriers to overcome for definitive characterization of such fields within a living cell [41]. Some of the concepts presented in this article need to be further characterized and validated; however, imaginative scientific ideas often lead to new discoveries. We are just beginning to understand the complicated nature of nuclear chromatin organization. The genomic landscape for transcriptional machineries within a living cell nucleus is more like an efficient metropolitan transportation system than a barren land sporadically dotted with meaningful transcription boot camps. Technologies such as single molecule live cell imaging, nucleic acid molecule beacons, and high throughput DNA sequencing, will provide powerful experimental support for us to explore transcriptional regulation circuits. New physical methods developed on the basis of nuclear magnetic resonance (NMR) technology may help us to explore the oscillation frequencies of different COCs within the cell nucleus, which would give us more insight into these oscillation coupling events in cell physiological and pathological states. High resolution characterization of the spatial and temporal transcriptional regulation of these chromatin oscillation clusters would improve our understanding of the dynamic nature of transcriptional regulation and the synchronized biological effects of different genes within a COC. With this knowledge we could manufacture small molecules that would be more efficiently recruited to specific genomic loci, for use in targeted gene therapy and drug delivery. More importantly, this knowledge would contribute materially to advances in stem cell research, developmental biology and cancer biology, which would finally address the question of how a single cell turns into a complicated human body at the molecular level [42,43].

## Competing interests

The authors declare they have no competing interests.

## Authors’ contributions

YZ conceived the general concepts and writes the article, QZ shared insights and gives advices to the article. Both authors read and approved the final manuscript.
